# Coil embolization of inferior epigastric artery pseudoaneurysm after percutaneous thrombin injection failure: a case report

**DOI:** 10.4076/1757-1626-2-6562

**Published:** 2009-08-12

**Authors:** Miltiadis Krokidis, Adam Hatzidakis, John Petrakis, Theodoros Lagoudis, Dimitrios Tsetis

**Affiliations:** 1 Department of Radiology, University Hospital of Heraklion, Medical School of CreteHeraklionGreece; 2 Department of General Surgery, University Hospital of Heraklion, Medical School of CreteHeraklionGreece

## Abstract

We report a case of a 71-year old woman with right inferior epigastric artery pseudoaneurysm following laceration by a computed tomography-guided 18G biopsy needle. The laceration was initially treated with placement of retained sutures; however the patient turned hemodynamically unstable 41 days later. Percutaneous ultrasound-guided injection of 1500 U of thrombin solution resulted in almost complete thrombosis of the pseudoaneurysm; however 24 hour control ultrasound revealed refilling of the pseudoaneurysm. Definite treatment was achieved by transcatheter coil embolization. Inferior epigastric artery pseudoaneurysm with underlying laceration may not respond to percutaneous thrombin injection, whereas coil embolization is shown to be effective.

## Introduction

Due to its anatomic location, inferior epigastric artery (IEA) laceration and pseudoaneurysm formation is a potential complication of removal of surgical retention sutures [1], paracentesis [2], catheter removal [3], drain placement [4] or percutaneous biopsy of abdominal masses [5]. Reported treatment choices include surgical repair [3,5], percutaneous techniques [1,2,4,6-8] or combination of both [9].

We report a case of iatrogenic pseudoaneurysm of the IEA which occurred 41 days after CT-guided biopsy of a mass lying under the abdominal wall and surgical tamponagè of lacerated IEA. Percutaneous thrombin injection failed to control the pseudoaneurysm and this was successfully embolized with coils. The efficacy of percutaneous thrombin injection for the treatment of iatrogenic pseudoaneurysms has been widely documented [8,10,11]. However only few reports exist on thrombin failure and to our knowledge this is the first reported case of thrombin failure in an iatrogenic IEA aneurysm treated definitely with coil embolization.

## Case presentation

**Figure 1.**
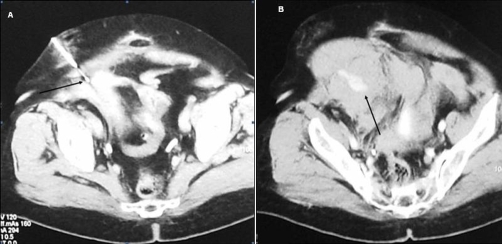
**(a)** Biopsy of a mass in the right inferior abdominal quadrant with an 18G Semi-automatic biopsy gun (arrow) **(b)** Hyperdense area (arrow) indicating active hemorrhage within the mass following 2nd biopsy puncture.

**Figure 2.**
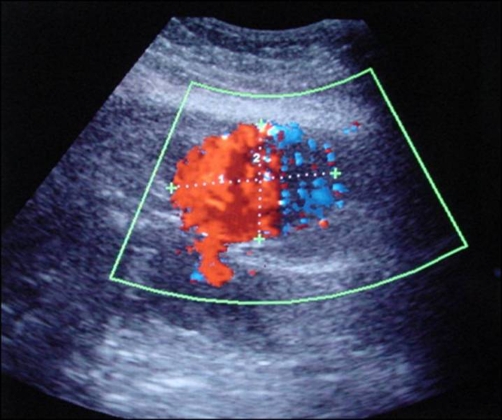
The 5.5 × 3.9 cm pseudoaneurysm 41 days after biopsy.

**Figure 3.**
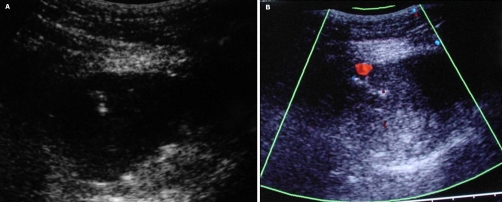
**(a)** Echogenic tip of the 21 G needle in the middle of the pseudoaneurysm cavity. **(b)** Almost complete thrombosis of the pseudoaneurysm after injection of 1500 U (1, 5 ml) of thrombin into the pseudoaneurysm sac.

**Figure 4.**
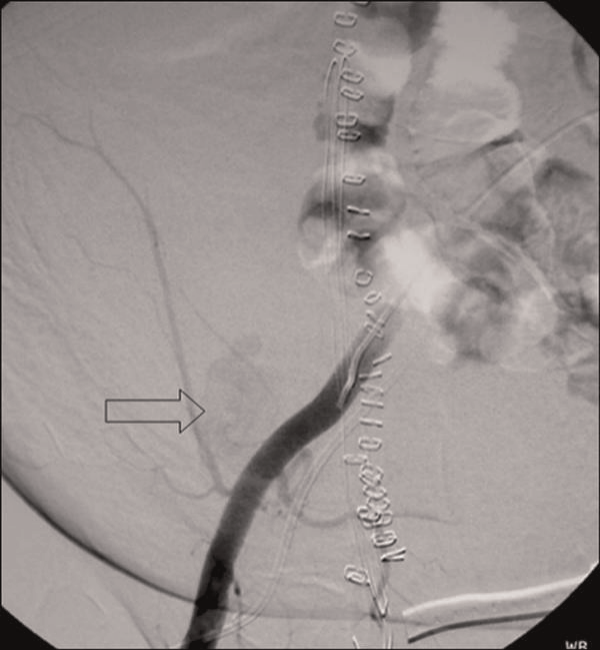
Selective angiography of the EIA demonstrates IEA pseudoaneurysm (arrow).

**Figure 5.**
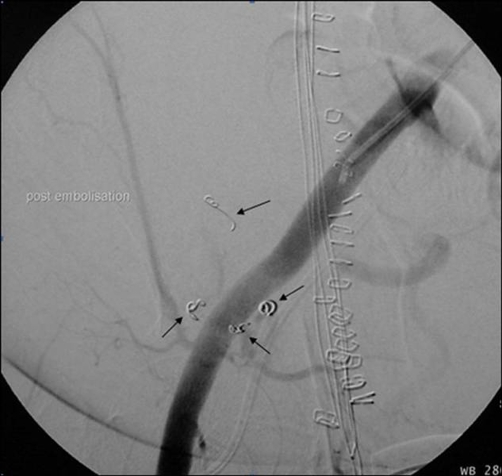
Successful embolization with metallic coils proximal and distal to the pseudoaneurysm neck.

A 71-year old Greek female patient with a history of surgical resection of gastrointestinal stromal tumor (GIST) from the jejunum 21 months ago, presented with a CT follow-up diagnosis of a mass in the right inferior abdominal quadrant in contiguity with the abdominal wall and the uterus fundus. In order to exclude recurrence, a CT-guided biopsy was decided. The biopsy was performed under local anesthesia with an 18G semi-automatic biopsy gun (Figure 1a). Immediately after the second puncture the patient became hemodynamically unstable and control scan revealed active hemorrhage within the mass (Figure 1b). The patient was immediately transferred to the operating theatre where surgical tamponnage with retention sutures around the punctured region was performed. She turned hemodynamically stable 7 days later and remained in the Intensive Care Unit for 21 days. The patient spent another 20 days in the General Surgery department for complete recovery. Forty-one days after the biopsy the patient suddenly became hemodynamically unstable again. She was transferred to the Interventional Radiology suite, where an US Doppler examination revealed the presence of a 5.5 × 3.9 cm pseudoaneurysm in the punctured region with a neck width of 0.58 cm (Figure 2). As this was considered as the cause of the hematocrit drop, treatment with percutaneous thrombin injection was decided. After local anesthesia a 21G needle was advanced under US-guidance and its tip was positioned in the middle of the pseudoaneurysm cavity away from the neck (Figure 3a). A solution of 1000 U/ml bovine-derived Thrombin Component (FloSeal, Baxter Healthcare, Fremont, CA, USA) was injected slowly at a rate of 0.1 ml/sec. The thrombotic process was controlled intermittently (in every 0.5 ml aliquots of the solution) with color Doppler. After a total amount of 1.5 ml of thrombin solution, almost complete pseudoaneurysm thrombosis was achieved (Figure 3b). No further thrombin injection was performed due to the patient’s hemodynamic instability. The patient’s hematocrit increased in the next six hours and she turned stable 12 hours later. The next day the patient complained of pain and hematocrit dropped again. An US examination revealed partial refilling of the pseudoaneurysm. Percutaneous embolization of the pseudoaneurysm was decided. Selective angiography of the internal iliac artery confirmed the presence of an IEA pseudoaneurysm (Figure 4). Superselective catheterization of the IEA with a 3Fr microcatheter (SP, Terumo Europe) and subsequent embolization with 5 mm metallic coils (Cook, Denmark) was performed successfully. The coils were deployed proximal and distal to the pseudoaneurysm neck, in order to stop blood supply through the superior and the inferior epigastric branches (Figure 5). The patient turned definitively stable and was released one week later. In the follow-up visit 6 months later no pseudoaneurysm recurrence was noted on US.

## Discussion

Inferior epigastric artery is a vessel keen to iatrogenic laceration after various medical procedures due to its superficial route. In the majority of cases a pseudoaneurysm is formed, the patient remaining stable. However, in one case of IEA laceration after percutaneous biopsy a large haematoma was formed, surgical control failed and patient died [5].

Surgical ligation of the lacerated vessel was not attempted in our case due to obscuration of the vessel by a large haematoma and therefore retention sutures were placed around the lacerated lesion. In a similar case reported by Todd et al after laparotomy, surgical access to the haematoma allowed direct ligation of the vessel. However the patient turned unstable and the patient died 36 hrs later [5]. We believe that the IEA pseudoaneurysm formation 41 days after the biopsy was probably due to the gradual absorbance of the formed haematoma or of one of the retention sutures which resulted in relaxation of the tamponating forces on the lacerated vessel.

Patients with IEA pseudoaneurysms may be hemodynamically stable or unstable, depending on the tamponating force that the extravasating blood receives from the surrounding tissues [1-4,10].

Treatment options of IEA pseudoaneurysms include surgical ligation of the lacerated vessel [3], transcatheter embolization [2,4,6], percutaneous thrombin injection [10] or direct compression [1].

Percutaneous thrombin injection is a widely accepted method for the treatment of pseudoaneurysms of the common femoral artery (CFA) that usually occurs after catheterization for interventional procedures [7]. There is only one case in the literature of a spontaneous IEA pseudoaneurysm which was treated successfully with percutaneous thrombin injection [8]. We decided to apply this type of therapy in our patient due to her debilitating condition.

Thrombin failure has been reported in the literature. Sheiman et al, in a study of 54 patients with simple CFA iatrogenic pseudoaneurysms treated with percutaneous US-guided thrombin injection concluded that their failure rate of 9% was due to an underlying sonographically occult vascular injury due to vessel laceration or infection. They also speculated that need for a dose more than 1000U of thrombin for pseudoaneurysm thrombosis is an indirect indicator of a large arteriotomy site defect requiring closer clinical follow-up [12].

In the reported case of IEA pseudoaneurysm that was treated with percutaneous thrombin injection, Shabani et al. refer a rare case of spontaneous pseudoaneurysm [8]. The authors do not refer previous traumatic mechanism or shear forces; therefore we may presume that the IEA has not been previously seriously lacerated. In our case 1500 U of thrombin were given but treatment failed for unknown reasons. A possible explanation may be that damaged endothelium at sites of a large arteriotomy or laceration expresses thrombomodulin which complexes with thrombin to activate protein C. Activated protein C is known to activate an anticoagulation pathway [13] and reduce the relative size and number of fibrin fibers within maturing thrombus [14]. This mechanism may help to explain why the clot which developed within the unsuccessfully treated pseudoaneurysm underwent spontaneous thrombolysis within 24 hrs leading to pseudoaneurysm recurrence.

Percutaneous coil embolization has shown to be effective in the hemorrhage control in all the reported cases [2,4,6]. In all cases the proximal and distal portions of the feeding vessel has been embolized with coils and complete thrombosis of the pseudoaneurysm has been achieved. Ferer et al, recommend surgical treatment for the cases of large pseudoaneurysms and percutaneous embolization for smaller lesions [15]. In a more recent study Lam et al, reported two cases of large pseudoaneurysms that have been successfully embolized [2].

We attempted initially the thrombin approach due to the fact it is a simple, quick and safe technique, minimally invasive, painless and very effective. We believe that the significant vessel wall laceration was the cause of the thrombin failure. Nevertheless, the patient was successfully treated with percutaneous embolization which is a more invasive, established intravascular therapeutic approach. We suggest this approach sequence as the most appropriate in the cases of IEA pseudoaneurysms.

## Abbreviations

CFA: common femoral artery
CT: computed tomography
GIST: gastrointestinal stromal tumors
IEA: inferior epigastric artery
